# Immune Mechanisms in Atherosclerosis, Especially in Diabetes Type 2

**DOI:** 10.3389/fendo.2013.00162

**Published:** 2013-10-29

**Authors:** Johan Frostegård

**Affiliations:** ^1^Unit of Immunology and Chronic Disease, Institute of Environmental Medicine, Karolinska Institutet, Stockholm, Sweden

**Keywords:** atherosclerosis, immune system, natural antibodies, phospholipids, inflammation

## Abstract

Atherosclerosis and ensuing cardiovascular disease (CVD) are major complications of diabetes type 2. Atherosclerosis is a chronic inflammatory condition involving immunocompetent cells of different types present in the lesions. Even though inflammation and immune activation may be more pronounced in atherosclerosis in diabetes type 2, there does not appear to be any major differences between diabetics and non-diabetics. Similar factors are thus implicated in atherosclerosis-associated immune activation in both groups. The cause of immune activation is not known and different mutually non-exclusive possibilities exist. Oxidized and/or enzymatically modified forms of low-density lipoprotein (OxLDL) and dead cells are present in atherosclerotic plaques. OxLDL could play a role, being pro-inflammatory and immunostimulatory as it activates T-cells and is cytotoxic at higher concentrations. Inflammatory phospholipids in OxLDL are implicated, with phosphorylcholine (PC) as one of the exposed antigens. Antibodies against PC (anti-PC) are anti-atherogenic in mouse studies, and anti-PC is negatively associated with development of atherosclerosis and CVD in humans. Bacteria and virus have been discussed as potential causes of immune activation, but it has been difficult to find direct evidence supporting this hypothesis, and antibiotic trials in humans have been negative or inconclusive. Heat shock proteins (HSP) could be one major target for atherogenic immune reactions. More direct causes of plaque rupture include cytokines such as interleukin 1β (IL-1β), tumor necrosis factor (TNF), and also lipid mediators as leukotrienes. In addition, in diabetes, hyperglycemia and oxidative stress appear to accelerate the development of atherosclerosis, one mechanism could be via promotion of immune reactions. To prove that immune reactions are causative of atherosclerosis and CVD, further studies with immune-modulatory treatments are needed.

## Background

Type 2 diabetes represents a major and growing problem throughout the world, not only in so-called developed countries. In addition to nephropathy and microvascular disease, cardiovascular disease (CVD), and accelerated atherosclerosis often occur in diabetes, both type 1 and 2 (1–3). The main focus of this review is immune activation in atherosclerosis, especially in type 2 diabetes.

The link between type 2 diabetes and inflammation is well established, and there are signs of chronic inflammation in both diabetes and insulin resistance (IR), a typical feature of type 2 diabetes (4). Also in atherosclerosis and CVD, chronic inflammation is a major feature, and in atherosclerosis, activated immune competent cells such as T-cells and antigen-presenting cells, are abundant in lesions (5).

Even though inflammation and size of the necrotic core may be increased in atherosclerosis in diabetes (6, 7), there was no difference in the prevalence of macrophages, lymphocytes, and overall inflammation in plaque or in the atherosclerotic cap between diabetics and non-diabetics according to the largest study in this area (8). It thus appears that there is no known fundamental difference between the immune activation and inflammation present in atherosclerosis among non-diabetics as compared to diabetics. Still macrophages and surface thrombi may persist longer after ischemic symptoms in diabetes, which could contribute to the increased risk of recurrent CVD in this condition (8) and risk factors as hyperglycemia naturally play a special role. In this review, I therefore discuss immune activation in atherosclerosis in general and in diabetes type 2 in the same context.

Acute inflammatory response developed from an evolutionary point of view most likely to protect against pathogens and to repair tissue damage, which could be caused also by trauma. The classic symptoms of acute inflammation – pain, swelling, redness, heat, and decrease of function – were described already in Hippocratic medicine. When acute inflammation is not resolved, but instead persists and becomes chronic, it can become a major problem. Indeed chronic inflammatory conditions represent a major disease burden in the western world, and increasingly, also in developing countries (9). Examples of chronic inflammatory diseases include rheumatic diseases such as rheumatoid arthritis (RA) and systemic lupus erythematosus (SLE); atherosclerosis and its major consequence, CVD including myocardial infarction (MI), acute coronary syndrome (ACS), claudication, and stroke; Alzheimer’s disease; diabetes type 2; increased IR and even abdominal obesity and osteoarthritis have inflammatory components (9).

Associations between these conditions are well known. For example, type 2 diabetes is a major risk factor for atherosclerosis and CVD (together with smoking, hypertension, dyslipidemia, age, and male sex) (10). Alzheimer’s disease and atherosclerosis and/or CVD have some risk factors in common (11) and smoking is a risk marker for RA in addition to well known effects in CVD (12). It has also become clear that there are associations between rheumatic diseases and atherosclerosis/CVD, especially in SLE (13). Also in RA, there is an increased risk of CVD according to many reports, and a recent meta-analysis imply that atherosclerosis *per se* is more prevalent in patients in RA (13–15). It is interesting to note that there are reports which also describe an increased risk of type 2 diabetes in RA (16).

Anti-inflammatory treatments have improved the prognoses of many patients in chronic inflammatory conditions, the most notable example being biologics such as tumor necrosis factor (TNF)-inhibitors in RA and other autoimmune conditions (9). There is therefore an apparent need to evaluate targeted anti-inflammatory and immunomodulative treatments in other chronic inflammatory conditions.

An interesting possibility would be that biologics such as TNF-inhibitors could be therapeutically effective in atherosclerosis and diabetes type 2 and their complications. However, this does not appear to be the case to any significant degree. Although systemic blockade of TNF has an anti-cachectic effect in RA patients, the data on anti-TNF effects of IR are conflicting, depending on disease severity and degree of inflammation (17–19). Still, a recent case report indicates that treatment with a novel T-cell inhibitor had a dramatic effect on IR in RA (20).

As discussed in an editorial (14), it is interesting to note that inflammatory nature of atherosclerosis was known already 180 years ago, reported by the famous Austrian pathologist K. Rokitansky. R. Virchow confirmed these findings somewhat later, and the ensuing debate between these two giants in the history of medicine is of interest also now (21, 22). Rokitansky argued that atherosclerosis is secondary to other disease processes and phenomena, while Virchow supported the view that inflammation in atherosclerosis is a primary pathogenic factor (21, 22). Both could be right, since atherosclerosis is nowadays recognized as an inflammatory process, and could be secondary to other inflammatory conditions.

A role of the immune system in atherosclerosis, with or without background of diabetes type 2, has been suggested since the 1980s, when activated T-cells were detected in human atherosclerotic lesions (23). Since then, an array of data indicate that immune activation is a major feature of and plays a role in atherosclerosis, and also that immunomodulation to ameliorate disease development could be an interesting possibility (10, 24, 25).

At an early stage of atherosclerosis, macrophages accumulate and become filled with lipids, mainly derived from modified forms of low-density lipoprotein (LDL). These lipid-filled macrophages develop into foam cells, and subsequently, these and other cells die, creating a necrotic core of cell debris. An organized apoptotic clearance, is thus not effective in advanced atherosclerotic lesions. Also lymphocytes, especially T-cells, are common at a very early stage of disease development. In the 1990s, it was demonstrated that immunomodulation can change the course of atherosclerosis development; while administration of heat shock protein 60/65 accelerated atherosclerosis development (26), immunization with oxLDL, had the opposite effect (27).

However, it should be noted, that there may be important differences between animal models and human disease in this context (28). Even though mouse models of atherosclerosis have very much increased our understanding of atherosclerosis it is still interesting to note that there may be problems with translating mouse data to humans. For example, lipid levels are strikingly much higher in mice models, and another problem is that it is difficult to mimic human CVD in animal models, including mice models (29, 30). In this review, I have therefore chosen to emphasize data on immunity and atherosclerosis which are derived from human studies, including *ex vivo* and cohort studies.

As discussed in a previous review (24), available evidence indicates that atherosclerosis *per se* is a normal part of human aging, though its complications may not be part of the normal aging process, at least not to the same extent.

A direct causative role played by T-cells is suggested by animal experiments, with transfer of beta(2)-glycoprotein I-reactive lymphocytes, which enhanced early atherosclerosis in LDL receptor-deficient mice (31). Further, CD4+ T cells reactive to modified low-density lipoprotein aggravate atherosclerosis (32). An immunomodulatory role of T-cells is suggested by experiments where regulatory T cells suppress immune activation and thereby inhibit atherosclerosis (33). Interestingly, also NK T-cells may play a role in this context since CD1d-dependent activation of NKT cells aggravates atherosclerosis (34). Relatively little is known about the role of T-cells in human atherosclerosis, but it is interesting to note that Th17/Th1 imbalances have been reported, which may be related to plaque rupture (35, 36). Interestingly, data in humans and mice provide support for the concept that TH17 cells induced upon TGF-b signaling promote the development of cap structures in atherosclerotic plaques and the role of T-cells could depend on the local activation pattern and milieu (37). Clearly, the role of T-cells in plaque rupture is complicated. Further, subsets of T-lymphocytes with pro-atherogenic and plaque-destabilizing properties are increased in diabetes type 2 and associated with a worse CVD-outcome (38).

During recent years, a more detailed picture also of other inflammatory and/or immune competent cells has emerged from *in vivo* and other experimental studies, which need to be corroborated in human disease.

One example is dendritic cells (DC) which are specialized antigen-presenting cells, which may play an important role in the initiation and progression of atherosclerosis (39). DC are present in immature forms in the arterial wall and become activated during atherogenesis. In human atherosclerosis, DC are present both at an early stage (40) and late stage, with higher numbers in vulnerable plaques (41). Similar findings have been reported in mice, where DC may promote atherogenesis (42). Further, DC and T-cells co-localize in plaques (43).

Monocytes/macrophages are present in lesions at different stages of atherosclerosis. Macrophages play a major role in inflammatory responses and may alter their phenotype which may vary on a scale, from pro-inflammatory M1 to anti-inflammatory M2 macrophages. Interestingly, even though the whole spectra of macrophages are present in lesions, including the relatively inert and surprisingly long lived macrophage-derived foam cells, M1 cells are prevalent in the vicinity of plaque rupture (44, 45).

Another cell type common in atherosclerotic lesions with inflammatory and potentially immune-modulatory properties is mast cells (46). A significant role played by this cell type in atherosclerosis and its complications is suggested by a recent report, where it is demonstrated that intraplaque mast cell numbers associate with future cardiovascular events (47).

Even though some B-cells, neutrophils, and NK-cells are present in lesions, it is not known to what extent they play a major role (10).

The role of the immune system in human atherosclerosis with or without background of diabetes type 2 is less defined as compared to the relevant animal models. T-cell reactive to OxLDL and related lipids are present in blood and atherosclerotic plaques (48, 49), patients with autoimmune diseases have increased atherosclerosis (13), and aspects of humoral immunity as natural antibodies against phosphorylcholine (PC) and other antigens are associated with atheroprotection (50). Humoral immunity has been shown to have pathogenic consequences in type 1 diabetes, triggered by immune complexes containing oxidized forms of LDL (51).

Available data thus imply that the immune system plays a major role in atherosclerosis, which could be seen as a chronic inflammatory disease or disease process. Interestingly, also diabetes, IR abdominal obesity, and the metabolic syndrome also have important inflammatory components.

## Diabetes and Causes of Immune Activation in Atherosclerosis

The key question herein is to elucidate what is the direct cause of the immune reactions in atherosclerosis, in general and in diabetes, and also how the inflammatory disease process can be influenced. There are several major hypotheses, non-mutually exclusive.

### Oxidation and other modifications of LDL and other moieties

Low-density lipoprotein can be modified by oxidation and/or enzymatic modification phospholipases being one example. LDL is also normally present in tissues as the intima of arteries, where it can bind to the proteoglycan matrix especially after modification. This binding is thought to be an early event in atherogenesis according to the “response to retention” hypothesis (52, 53).

Oxidized low-density lipoprotein has pro-inflammatory and immune-activating properties, activating endothelial cells, monocytes/macrophages, and T cells (49, 54, 55). OxLDL also toxic at higher concentrations and an important feature of atherosclerotic lesions, perhaps somewhat understudied, is the abundance of dead cells. It is thus possible that OxLDL is one cause of such cell death (49, 54, 55). Enzymatically modified LDL could play a major role, and PLA2, which causes such modification, is expressed in both normal arteries and atherosclerotic lesions (56) and can induce activation of DC (57). Inflammatory phospholipids such as lysophosphatidylcholine (LPC) and/or platelet activating factor (PAF)-like lipids cause much of OxLDL:s effects which can occur through the PAF-receptor (58–61) or other mechanisms including Toll-like receptor- and scavenger receptor-interaction (62, 63).

In general oxidized phospholipids (OxPL) are implicated in immune reactivity in atherosclerosis, and could be derived from LDL-modification but also from cell membrane changes. Such oxPL include LPC, and often, a shortened sn-2 position in the fatty acid moiety serves as a danger-associated molecular patterns (DAMP). Oxidation turns OxPLs into markers of modified self, which are recognized by both soluble and cell bound receptors such as scavenger receptors, natural antibodies, and also C-reactive protein (CRP). The common theme in these different system is likely to be removal of senescent and dead cells, but also oxidized or otherwise modified lipoproteins. This has been described in several recent publications (62, 64–69).

Another important example of DAMP, in addition to PC and oxPL epitopes, is malonyl-dialdehyde (MDA) which is also generated during LDL-oxidation. MDA forms adducts on proteins, carbohydrates, and DNA (63).

Other compounds which could be implicated as atherogenic in OxLDL are modified and/or oxidized forms of apoB and cholesterol. Such modified compounds may play a role but the underlying mechanisms need to be better defined (63, 70). Clinical studies support the hypothesis of inflammatory phospholipids as causes of atherosclerosis, whereas levels of OxLDL are raised in the metabolic syndrome (71), in hypertension (72) and in established type 2 diabetes (73). Further, high levels of MDA-LDL in isolated immune complexes predict future MI and acute CVD events in patients with type 2 diabetes (74).

Many epidemiological studies demonstrate that smoking is associated with atherosclerosis and CVD (75, 76) and animal experiments demonstrate that smoking promotes atherogenesis (77–79). Somewhat surprisingly, underlying mechanisms are not fully clarified. Still, one mechanism is smoking-induced increased lipid-oxidation (80) and closely related to this, oxidative stress (81). Smoking is associated with systemic and local inflammation with raised levels of pro-inflammatory cytokines and cells, in particular in chronic obstructive pulmonary disease (82), which could play a role also in atherosclerosis.

Additionally, several factors appear to be diabetes-specific and able to further aggravate atherosclerosis (and CVD) among diabetics. One major such factor is reduction of endothelial nitric oxide (NO)-levels, leading to deterioration of endothelial function, an early sign of vascular problems and increased risk of atherosclerosis and CVD. Hyperglycemia *per se* leads to increased levels of reactive oxygen species, which in turn inactivate NO and thus impair endothelial function. One common denominator is the formation of advanced glycation end products (AGEs) which have pro-inflammatory and potentially atherogenic properties (83–88). Interestingly, atherosclerosis is suppressed by the soluble receptor for AGEs in an animal model (89). Also susceptibility of LDL and its subfractions to glycation could play a role, in diabetes type 2 (90) AGE-products of LDL are able to generate autoantibodies, which on the other hand appear to have pro-inflammatory properties (91). Another interesting connection between AGEs and OxLDL are previous findings where AGEs initiate LDL oxidation (92). Further, increased oxidation of LDL in diabetes could promote atherogenesis (73, 93).

Other potential underlying factors are present in type 2 diabetes, but where the connection to immune reactivity in atherosclerosis is less clear, and thus not a focus herein. These include increased circulating free fatty acids in patients with abdominal obesity, which could be atherogenic by decreasing HDL-levels, increasing levels of small dense LDL and also by a negative effect on endothelial function (3, 94). Other mechanisms include effects on smooth muscle cells, such as induction of apoptosis and thus plaque instability (95) and also a hypercoagulative state by alterations in platelet function (96).

### Autoantibodies and immune complexes related to phospholipid epitopes and OxLDL

An interesting possibility is that immune complexes containing OxLDL could contribute to vascular damage by promoting inflammation and atherosclerosis, which has been reported in type 2 diabetes (97, 98). In addition to oxLDL, also MDA-LDL and AGE-product-modified LDL induce immune responses in humans (99). The role of antibodies against these different forms of modified LDL is less clear, and varies in different studies: both negative and positive associations have been described. This could depend on different methods used, different degrees of oxidation and also on different immunoglobulin subclasses and isotypes. On the other hand, the antigenic constitution of immune complexes formed with modified forms of LDL and their corresponding antibodies may influence their pathogenicity, as suggested by the recent observation that high levels of highly oxidized forms of LDL (MDA-LDL) in isolated IC predict future MI and acute CV events in patients with type 2 diabetes (74).

It is also possible that there are differences between mouse and man which could be the basis for the conflicting views about the pathogenic or protective role of the antibody response to modified forms of LDL (100, 101).

Another type of antibodies that has attracted attention are those against PC (anti-PC), which are often considered as “natural” antibodies. We have reported in several publications that anti-PC is a protection marker for atherosclerosis development and CVD, in different populations, both healthy individuals, ACS patients, and patients with SLE or RA (50, 102–107). Animal experiments also support a protective role of anti-PC. Immunization with pneumococci containing PC induced a decrease in atherosclerosis development in a mouse model in parallel with an increase in anti-PC, among other antibodies (108). Passive and active immunization raising anti-PC levels decrease atherosclerosis in mouse models (109, 110).

Human anti-PC could be anti-atherogenic and decrease risk of CVD by anti-inflammatory effects (107), inhibition of oxLDL-uptake through scavenger receptors (111) and inhibition of LPC-induced cell (112). In mouse models, the anti-inflammatory effect of anti-PC was confirmed, and facilitating phagocytosis was reported as one mechanism. Anti-PC antibodies bind dead and dying cells, enhancing their phagocytosis and clearance (113). Further investigations are necessary to determine whether the data generated in animal models translates to human medicine.

A Western life style could play a role to influence anti-PC levels, and one underlying factor could be infections which are not prevalent in developed countries (114, 115). Gluten in the diet could also play a role being a novel component of human diet from an evolutionary point of view (116). The heritability of anti-PC is 37%, allowing also for genetic factors (117). In line with these findings, a recent report indicates that antibodies against reactive a-dicarbonyls such as methylglyoxal (MGO) are negatively associated with atherosclerosis development among patients with diabetes type 2. This finding suggests an additional role played by diabetes type 2 immune reactivity in atherosclerosis development as compared to the general population (118). Still, it is interesting to note that MGO-LDL is only weakly immunogenic in humans (119). Clearly, further research is needed to clarify the role by AGE-recognizing antibodies in diabetes type 2 and atherosclerosis.

A potential role of epigenetic changes in both atherosclerosis/CVD and type 2 diabetes is not the topic of this review, though a very interesting subject. One can not exclude that epigenetic mechanisms may influence immune mechanisms in both these conditions. In an intriguing study, it was recently demonstrated that in an atherosclerosis mouse model, maternal immunization with oxidized LDL affects *in utero* programing of both atherosclerosis and IR and type 2 diabetes, promoting protection against development of these conditions (120) Whether this can be translated to humans also remains to be demonstrated.

### Heat shock proteins

Antibodies against heat shock proteins (HSPs), especially HSP60/65 but also others like HSP70 and HSP90 have been described as potential causes of atherosclerosis and CVD. HSPs are immunogenic, and T-cell clones recognizing HSP60 are present in atherosclerotic plaques (121, 122). HSPs may activate immune reactions through cross-reactivity with HSP from microorganisms as bacteria. This is supported by both clinical data with associations between antibodies against HSP60/65 and atherosclerosis, and experimental data where immunization with HSP 60/65 increases atherosclerosis in an animal model (26, 123).

We hypothesized that hypertension possibly could cause and immune reaction and inflammation in arteries by induction of HSP 60/65, which are also induced by oxLDL (124, 125) and in principle, in conditions such as diabetes, where LDL-oxidation is implicated, this could further enhance the progression of atherosclerosis.

### Infections

Infections have since long been hypothesized to be a cause of atherosclerosis. Many pathogenic candidates have been proposed, one not excluding another. *Chlamydophila pneumoniae* (CP); periodontal organisms including *Porphyromonas gingivalis* (PG) and *Aggregatibacter actinomycetemcomitans* (AA); *Helicobacter pylori* (HP) and Cytomegalovirus (CVM) are among the most promising candidates, since they are present in plaques, promote atherosclerosis in animal studies, and have associations with disease in humans (126).

Early studies demonstrated presence of CP in atherosclerotic plaques (127) and that antibodies against CP were associated with CVD (128–130) but later also negative studies were published (126).

However, treatment with CP-targeting antibiotics had no effect on atherosclerosis (131–133). This clearly argues against CP as a causative agent, though there could be other explanations, for example, CP is difficult to reach within the plaque and thus it is still possible that at the earlier stages of CP-infection antibacterial treatment could be beneficial (126).

Periodontal pathogens, as PG and AA, are also interesting pathogenic candidates. The association with periodontitis and CVD/atherosclerosis has been debated since there are confounders, including social ones, which may be difficult to control for. A recent statement from the American Heart Association supports an independent association between periodontal disease and atherosclerosis, but available data do not prove causation, even though it is interesting that intervention does decrease systemic inflammation and improves endothelial function (134).

Still it is interesting that periodontitis and diabetes could be related to each other, and in principle, periodontitis could be diabetogenic and then also increase the risk of atherosclerosis and CVD, while diabetes could increase the risk of periodontitis (135–137).

An inherent problem with viral infections such as CMV from the Herpes virus group is that they are very common, thus making the interpretation of any associations with diabetes, atherosclerosis or CVD complicated. It may be that certain patients subgroups that are prone to accelerated atherosclerosis and CVD, as it is seen for example after organ transplantation, CMV infection plays an important role (138). CMV is present in atherosclerotic lesions in many but not all studies (126), but also in healthy arteries (139) so in principle CMV could be just an innocent by-stander. However, CMV induces migration of arterial smooth muscle cells *in vitro*, suggesting potential pro-atherogenic mechanisms (140).

*Helicobacter pylori* infection, causing gastritis and gastric ulcer, may be implicated as supported by indirect evidence, where reduction of CVD after eradication of HP was reported (126, 141). However, no viable HP had been isolated from atherosclerotic plaques, and mouse experiments in general do not support a pathogenic role of HP in atherosclerosis (142).

Other pathogens including HIV, EBV, influenza virus, *Mycoplasma pneumoniae* and *Streptococcus pneumoniae* have also been discussed, but as of yet there is no conclusive causative evidence available (126). Borreliosis caused by spirochetes, is independently associated with CVD, although little is known from atherosclerosis-related experimental studies (143).

It is not clear if infections on the diabetic background contribute more to atherosclerosis, but it is interesting to note that an increased rate of infections with *Chlamydia pneumoniae* has been reported in patients with diabetes type 2 (144).

Taken together, even though the infection hypothesis in atherosclerosis with or without diabetes type 2 is very interesting and is supported by circumstantial evidence, there is still little direct evidence of a potential causative or pathogenic role of microorganisms in CVD/atherosclerosis. However, we can not exclude a possibility that infectious agents act in concert with diabetes-specific factors to promote atherosclerosis.

Infections could also be of importance indirectly. Many pathogens are present in lesions, and could start or promote an ongoing local inflammatory process which could lead to increased atherosclerosis and, in principle, also to CVD and plaque rupture – at such sites, the local inflammation appears to be especially strong (5). Also the total infectious burden is associated with increased atherosclerosis and CVD, and the risk of infection is raised in diabetes, especially when blood glucose levels are not well controlled. Platelet aggregation and endothelial dysfunction, could also influence atherogenesis in this context (126, 145).

### Other types of immune activation in diabetes type 2 and atherosclerosis

Another potential link between atherosclerosis and diabetes type 2, and thus also hyperglycemia and metabolic dysfunction, is provided by a recent study, where NKG2D, an immune-activating receptor expressed by different types of immune cells was tested (146). The authors reported that blocking NKG2D in apolipoprotein E-deficient (apo E^−/−^) mice led to a dramatic reduction in plaque formation, suppressed systemic and organ-specific inflammation, and improved abnormal metabolic conditions. Thus the NKG2D/ligand interaction could drive both: inflammation related to metabolic dysfunction/diabetes type 2 and atherosclerosis. Further, the molecules and pathogens discussed earlier such as OxLDL, HSPs, AGEs, and infectious agents, could upregulate NKG2D on different cell types, which in turn can activate T-cells, NK, and NKT cells and thus promote atherosclerosis; also other cell types as endothelial cells could produce pro-atherogenic factors, including cytokines (146).

It is well known that inflammation is a link between IR, obesity, and diabetes. Further abdominal obesity and IR are classic features of type 2 diabetes (147).

There are many examples of links between inflammation in other conditions and atherosclerosis. Periodontitis is also a systemic inflammatory condition, which is more frequent in patients with diabetes, could increase the risk of atherosclerosis and CVD also indirectly (137).

Raised levels of CRP is a risk marker for atherosclerosis and CVD in many studies, though it is not clear if CRP is either protective or detrimental for disease development. Cytokines as IL-6 and raised systemic levels of OxLDL are other examples of pro-inflammatory molecules that are associated with increased risk of CVD and atherosclerosis (10). Another group of inflammatory compounds that are present in advanced atherosclerotic plaques are lipid mediators, such as leukotrienes (148, 149).

Another example of associations between systemic inflammation and atherosclerosis and CVD is a number of autoimmune diseases. The evidence is strongest for SLE, where the risk of CVD is very high (13, 14, 150). Also in RA and other types of autoimmune conditions, atherosclerosis is increased according to many studies including a recent meta-analysis (13–15).

## Treatment Against Inflammation and Immune Activation to Ameliorate Atherosclerosis, Decrease Risk of CVD, Especially in Diabetes

### Optimal diabetes treatment

Since diabetes type 2 is an inflammatory condition, first and foremost, control of the disease itself is likely of major importance for amelioration of pro-atherogenic inflammation and immune reactions. The specific optimal range of glycemic control in relation to CVD and atherosclerosis is still debated (151). This is similar to the situation in rheumatic diseases, where at least for RA, the risk may be similar to that in type 2 diabetes and where disease controls appears to be of importance (13).

### Statins

Statins were developed to treat hyperlipidemia and are often used in diabetes, though there is still some controversy about their exact role in this condition (152). Interestingly, statins have additional anti-inflammatory effects rather than simply decreasing cholesterol levels, which could be of major importance in atherosclerosis and CVD, not least in diabetic patients. These properties could be seen as “side-effect” of HMG CoA-reductase inhibition. Interestingly, statins have immunomodulatory properties, such as impairment of CD1d-mediated antigen presentation through the inhibition of prenylation (153) and decreasing MHC class II interaction with antigen (154). Further, the lipid-lowering effect of statins could by itself influence the immune reactions to LDL-related antigens, since a decrease in LDL-levels is likely to decrease also exposure of the immune system to modified forms of LDL, thus reducing the intensity of the corresponding immune response.

The Jupiter study demonstrated that statin treatment may be beneficial for individuals with increased high sensitivity CRP but normal LDL and it is possible that the beneficial effects of statins to some extent are in fact caused by their anti-inflammatory and immune-modulatory properties (155, 156).

### Anti-inflammatory treatment

Even though the major focus of this review is immune activation it is still interesting to discuss this in the context of inflammation in general, especially in relation to treatment. There is as yet no established anti-inflammatory treatment of atherosclerosis or CVD, with or without diabetes. There are several interesting possibilities that are currently being investigated. In RA, treatment with methotrexate weekly is a very common schedule, and a recent meta-analysis demonstrate that the risk of CVD is decreased in patients with RA treated with methotrexate (157). Animal studies show that methotrexate decreases atherogenesis (158). In the cardiovascular inflammation reduction trial (CIRT) low dose methotrexate (target dose 20 mg/week) is tested for reduction of CVD events among post-MI patients with diabetes or metabolic syndrome (156). There are different opinions about whether biologics as anti-TNF are beneficial from a cardiovascular point of view, though a recent study where decrease of CVD was reported in RA adds support to this possibility (159, 160). The Canakinumab Anti-Inflammatory Thrombosis Outcomes Study (CANTOS) investigates interleukin-1β (IL-1β) inhibition and reduced risk of MI, stroke, and CVD in stable coronary artery disease patients with persistent elevations of CRP (≥2 mg/L) (156, 161).

Other interesting anti-inflammatory treatments could be inhibition of inflammatory lipid mediators as PAF (162). Annexin A5 is anti-thrombotic plasma protein, which is anti-inflammatory, inhibits atherosclerosis development and improves endothelial function in a mouse model (163). Annexin A5 could thus be a possible therapy candidate. Inhibition of phospholipases as treatment in patients after ACS are currently in trials (164).

### Immunomodulatory therapy

Studies in mid-90s demonstrated that immunization with modified forms of LDL ameliorated atherosclerosis in animal models (27), providing initial evidence for immunomodulation as a potential treatment against atherosclerosis. One line of treatment is to target the apoB components which decreases atherosclerosis development in animal models (70, 165). However, such treatment did not show positive expected effect in humans (166), though the possibility that the end points used in this study where not optimal cannot be excluded.

Another possibility is that the phospholipid moiety on OxLDL could be the basis of immunomodulation, one example being PC. This is supported by clinical studies, animal and *in vitro* experiments (50). As discussed, mechanisms include anti-inflammatory (107, 167), inhibition of cell death (112), and decreased uptake of oxLDL in macrophages (105). Further, administration of immunoglobulins has shown promising results in animal studies (168). Another interesting possibility is to ameliorate atherosclerosis by modulating immune reactions against HSP (169, 170).

## Summary and Conclusion

Taken together, atherosclerosis and ensuing CVD represents major health problems in the developed world and especially so in patients with diabetes, where macro-vascular complications is a common problem. It has become clear that atherosclerosis on a background of diabetes or without is a chronic inflammation characterized by presence of activated immune competent cells throughout the lesions. Atherosclerosis in diabetes, though accelerated, does not seem to be very different from atherosclerosis in individuals without diabetes type 2, although certain diabetes-specific factors could contribute to pro-atherogenic immune activation and thus aggravate atherosclerosis and risk of CVD. In diabetes, reduction of endothelial NO-levels, systemic hyperglycemia if uncontrolled, generation of reactive oxygen species, oxidative stress, and increased LDL-oxidation, formation of AGEs, and increased circulating free fatty acids are factors that add to atherosclerosis and CVD risk. Potential triggers of immune activation in atherosclerosis in the general population (as well as among diabetics) include OxLDL and other LDL modifications and corresponding antibodies, possibly infections, high levels of heat shock protein antibodies, and low levels of natural antibodies as anti-PC. Clinical trials and other studies of immune-modulatory and anti-inflammatory treatment in atherosclerosis and CVD are currently conducted but until such treatments are proven to be effective in humans, the exact role of immune reactions and inflammation in human atherosclerosis with or without type 2 diabetes remains to be clarified.

## Conflict of Interest Statement

I am named as inventor on patent and patent applications relating to immunity and phospholipid antigens.
